# Machine learning modelling for predicting the utilization of invasive and non‐invasive ventilation throughout the ICU duration

**DOI:** 10.1049/htl2.12081

**Published:** 2024-02-20

**Authors:** Emma Schwager, Mohsen Nabian, Xinggang Liu, Ting Feng, Robin French, Pam Amelung, Louis Atallah, Omar Badawi

**Affiliations:** ^1^ Philips Research North America Cambridge Massachusetts USA; ^2^ Philips Clinical AI and Analytics New Brunswick New Jersey USA; ^3^ Johnson and Johnson Limited New Brunswick New Jersey USA; ^4^ Philips EMR & Care Management Cambridge Massachusetts USA; ^5^ Trial Library San Francisco California USA

**Keywords:** medical computing, medical information systems, medical signal processing, patient care, patient monitoring, pattern classification

## Abstract

The goal of this work is to develop a Machine Learning model to predict the need for both invasive and non‐invasive mechanical ventilation in intensive care unit (ICU) patients. Using the Philips eICU Research Institute (ERI) database, 2.6 million ICU patient data from 2010 to 2019 were analyzed. This data was randomly split into training (63%), validation (27%), and test (10%) sets. Additionally, an external test set from a single hospital from the ERI database was employed to assess the model's generalizability. Model performance was determined by comparing the model probability predictions with the actual incidence of ventilation use, either invasive or non‐invasive. The model demonstrated a prediction performance with an AUC of 0.921 for overall ventilation, 0.937 for invasive, and 0.827 for non‐invasive. Factors such as high Glasgow Coma Scores, younger age, lower BMI, and lower PaCO2 were highlighted as indicators of a lower likelihood for the need for ventilation. The model can serve as a retrospective benchmarking tool for hospitals to assess ICU performance concerning mechanical ventilation necessity. It also enables analysis of ventilation strategy trends and risk‐adjusted comparisons, with potential for future testing as a clinical decision tool for optimizing ICU ventilation management.

## Graphical abstract

This study developed a Machine Learning model using the Philips eICU Research Institute database to predict the need for invasive and non‐invasive mechanical ventilation in intensive care unit (ICU) patients. Analyzing data from 2.6 million ICU patients from 2010 to 2019, the model achieved high accuracy in performance, indicated by AUC values. Notably, factors such as Glasgow Coma Scores, age, BMI, and PaCO2 influenced the likelihood of ventilation necessity.

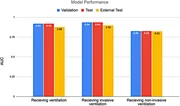

## 1 INTRODUCTION

Ventilation is a critical part of intensive care unit (ICU) patient care. Mechanical ventilation has proven to be a lifesaving intervention for patients in respiratory distress and delays in intubation may carry significant risk [1, 2]. Although it is vital to provide mechanical ventilation support promptly for patients who need it, its use comes with some risks, such as permanent lung injuries [3] and infection. Its use can lead to ventilator‐associated complications which can include mortality in some cases [4]. Non‐invasive ventilation is increasingly used to supplement or mitigate the use of invasive mechanical ventilation [5, 6].

Understanding which patients in an ICU patient population are likely to need ventilation can help ICUs optimize their ventilation management approach at an institutional level. Such benchmarking approaches have been developed for other key patient management outcomes such as ICU mortality [7]. Methods that can risk adjust the probability of use of each type of ventilation, would help evaluate the changing patterns of ventilation over time. An example is the evaluation with more certainty if the same types of patients are more likely to be treated with non‐invasive ventilation than in prior years.

Previous approaches to predicting patients’ need for ventilation have focused largely on predicting the need for invasive ventilation specifically [8, 9], or on predicting the need for ventilation for particular sub‐groups of patients [10]. In this work, we developed the Philips Critical Care Outcome Prediction Model for Ventilation Probability, a novel algorithm for predicting the need for ventilatory support for patients during their ICU stay, using data from ICU admission. The algorithm also predicts the *type* of ventilation needed: invasive or non‐invasive ventilation. This algorithm would allow institutions to compare the breakdown of their ventilated patients against institutions that admit similar groups of patients.

## 2 METHODS

### 2.1 Study population

The eRI (eICU Research Institute) database, housing all historical data collected from participating Philips’ tele‐critical care programs, was used for the model development and validation. The eRI database captures all patient unit stays admitted to ICUs for health systems that agreed to participate in the database. Data captured includes parameters monitored by the Philips eICU program with raw physiologic, diagnosis, and treatment information [11]. The eICU data was determined by experts at Privacy Analytics to be de‐identified under Health Insurance Portability and Accountability Act (HIPAA) and is exempt from Institutional Review Board (IRB) oversight [12]. The algorithm was trained using the eRI database with patients admitted to 350 hospitals between 2010 and 2019. The database includes a variety of unit types (such as medical, surgical, cardiac, neuro, and trauma) from ICUs across the United States, in rural and urban areas as well as teaching and non‐teaching hospitals.

Using this database, 3,818,428 de‐identified ICU patient stays from 350 hospitals between 2010 and 2019 were included in the total cohort. For each patient, we used the records to identify invasive ventilation as any ventilation involving the use of inserting tube into the patient's airway. Similarly, we identified non‐invasive ventilation when no inserting tube in the airway was used, but rather ventilation was performed with non‐invasive techniques such as the use of mask on the nose and face (ex. BiPAP). Consistent with similar works, such as Acute physiology and chronic health evaluation (APACHE) [13], the use of continuous positive airway pressure (cPAP) was not considered as any type of mechanical ventilation. A more detailed explanation on the definition and extraction of invasive and non‐invasive ventilation data are provided in the supplementary section. We excluded patients younger than 16 years old, patients with ICU stays less than 4 h as well as patients with unclear ventilation outcome. We also excluded patients with missing values for the required variables of the model (Appendix Tables 1A and 2A). The final 2,655,445 stays were divided into 63% for training, 27% for validation, and 10% for unseen test set. To further examine model's generalizability to institutions outside the training pool, we also evaluated our model on an external test set consisted of a single hospital with 45K stays over the study period (2010–2019).

### 2.2 Data extraction and definitions

Demographic and admission characteristics, vital signs, and laboratory measurements were extracted. Categorical variables such as admission source or admission diagnosis were converted to binary variables for each category (1 if present, 0 if not). Vital signs and laboratory measurements were summarized (using mean or mean and variance) over the first 24 h of ICU stay. If no data was available during the first 24 h, the summary window was extended to the 6 h prior to ICU admission. Less‐commonly measured continuous variables such as lactate or pH were converted to categorical variables, including a ‘missing’ category.

### 2.3 Model training and evaluation

**FIGURE 1:**
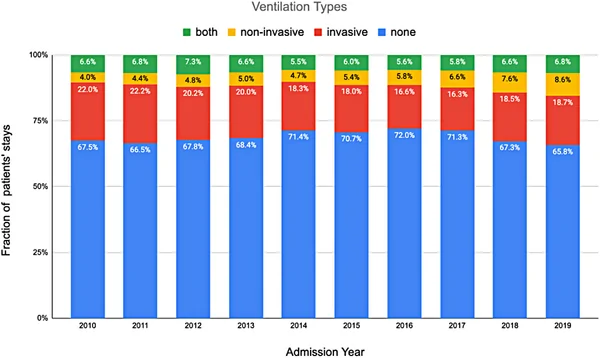
The prevalence of the ventilation use over time from 2010 to 2019. The use of non‐invasive ventilation seems to increase over time while the invasive ventilation use seems to slightly decrease.

A multiclass gradient boosting model was trained to predict whether a patient belonged to one of four classes of ventilation status at any time during their ICU stay (Figure 1): none (receiving neither invasive nor non‐invasive ventilation); non‐invasive‐only (receiving only non‐invasive ventilation but not invasive ventilation); invasive‐only (receiving only invasive ventilation but not non‐invasive ventilation); and both (receiving both invasive and non‐invasive ventilation).

**FIGURE 2:**
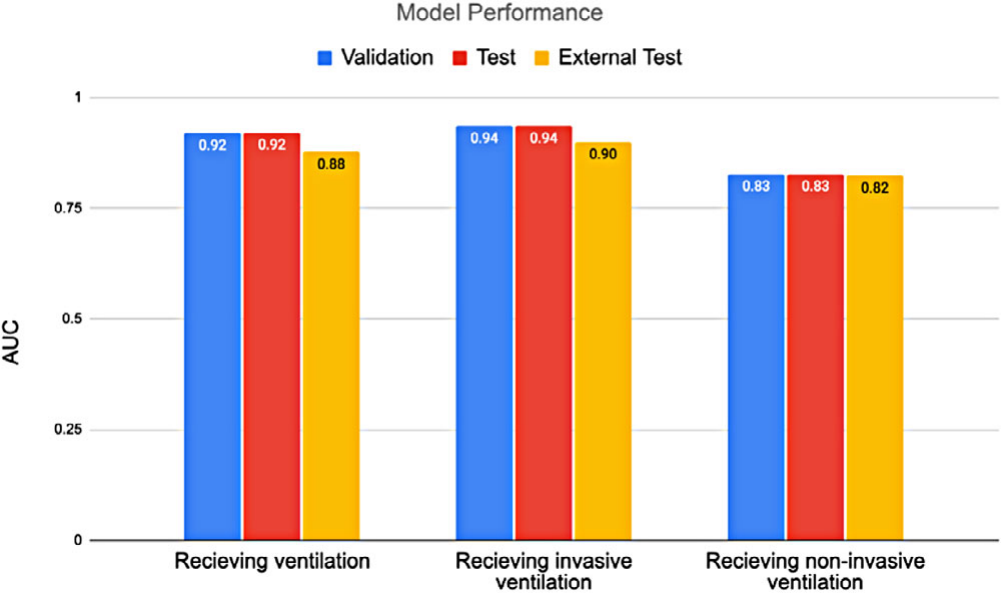
Model performance. The model's performance for identifying patients receiving any ventilation (left), patients receiving any invasive ventilation (middle), or patients receiving any non‐invasive ventilation (right).

**FIGURE 3:**
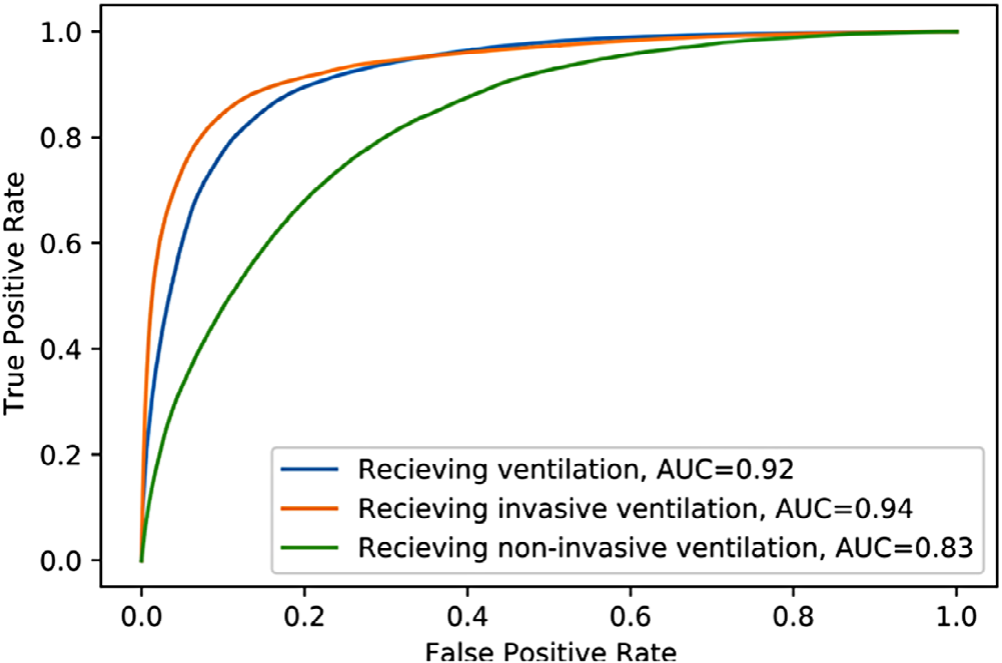
ROC curve. The model's performance on test data for identifying patients receiving any ventilation (blue), patients receiving any invasive ventilation (orange), or patients receiving any non‐invasive ventilation (green). ROC, receiver operating characteristic.

**FIGURE 4:**
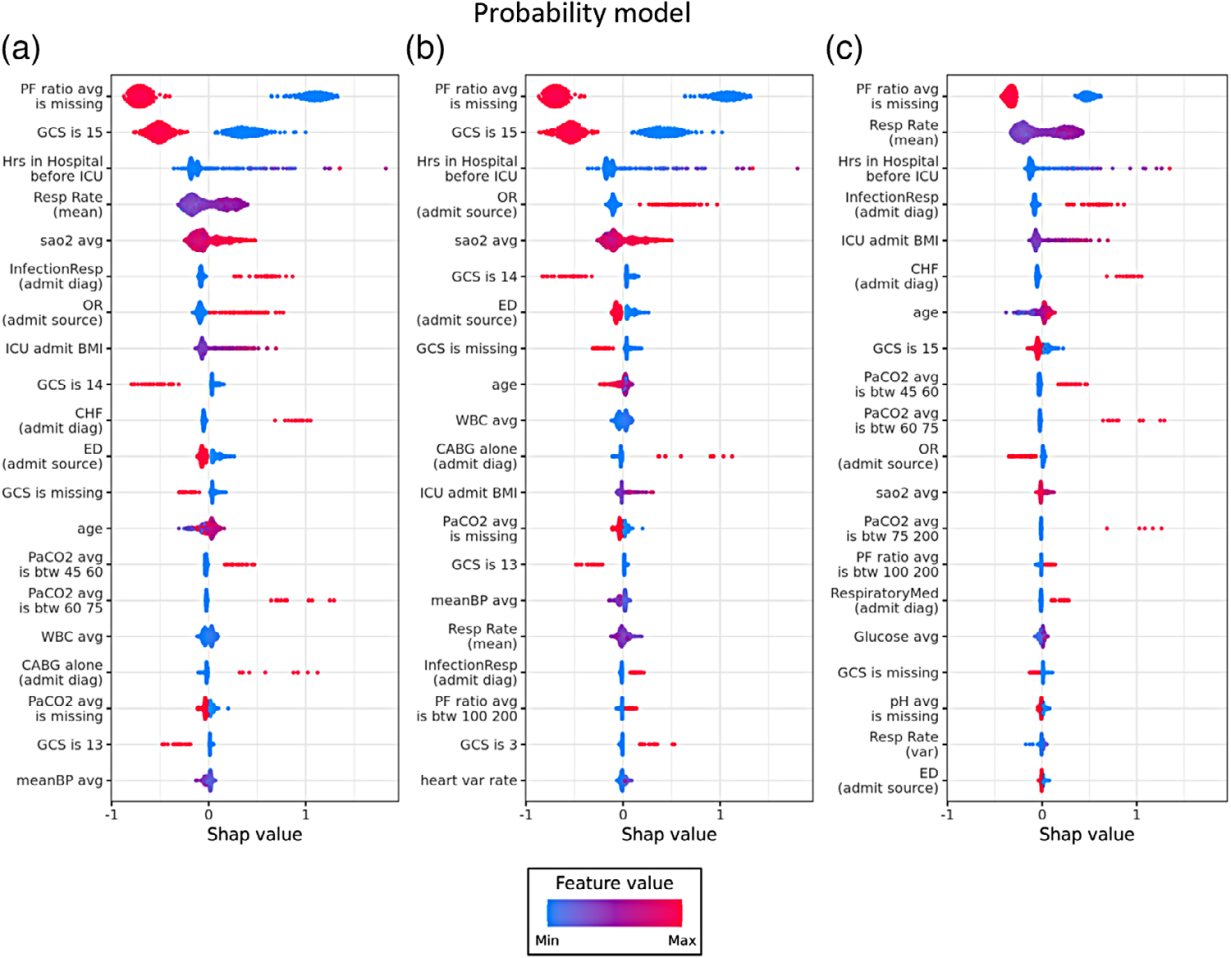
The top 20 features contributing to model predictions. The top 20 features for predicting any ventilation (a), any invasive ventilation (b), or any non‐invasive ventilation (c). Each point represents a single stay. The colour represents the feature value for that stay: from the minimum (bright blue) to the maximum (bright red). The *x*‐axis indicates the SHAP value: positive values correspond to increased likelihood of receiving ventilation during the stay, and negative values to decreased likelihood. SHAP, shapley additive explanations.

Although the model was trained with four classes, for direct utility, outcome predictions are modified for three predictive categories: any ventilation versus no ventilation; any invasive ventilation versus no invasive ventilation; and any non‐invasive ventilation versus no non‐invasive ventilation. The predicted probabilities were calculated by summing the predicted probabilities for the component classes. Model performance was quantified using the receiver operating characteristic (ROC) curve (Figure 3) and the area under the ROC curve (AUC) (Figure 2). Feature importance was evaluated using SHAP (Shapley Additive exPlanations) values [14] (Figure 4).

## 3 RESULTS

### 3.1 Cohort characteristics

We examined the demographic and patient outcome breakdowns among the four ventilation outcomes (Table 1). Most patients (68%) did not receive any type of ventilation. Of those who did, most (18%) received only invasive ventilation, while there was a nearly equal split between those receiving non‐invasive ventilation alone and those who received both types (8% non‐invasive‐only vs. 6% both). Patients who did not receive ventilation tended to have shorter ICU stays and lower mortality rates, while patients who received both types of ventilation had much longer ICU stays and higher mortality rates. Patients receiving only non‐invasive ventilation tended to be older (average age of 68) and were more likely to be female compared to the other three outcomes.

**TABLE 1:** The distribution of patient outcomes and demographics by the type of ventilation received.

	None	Invasive only	Non‐invasive only	Both
# ICU stays	1,133,194 (62.8%)	442,729 (24.6%)	131,759 (7.3%)	95,381 (5.3%)
Age (y)	62.78 (17.72)	61.85 (16.47)	68.59 (14.25)	64.97 (14.46)
Admit BMI	28.43 (7.68)	29.05 (8.01)	31.28 (10.46)	31.2 (10.01)
Female^*^	530,885 (46.8%)	184,631 (41.7%)	66,271 (50.3%)	42,357 (44.4%)
Hospital LOS (days)^#^	4.29 (4.81)	7.48 (8.75)	6.75 (6.71)	12.09 (13.15)
ICU LOS (days)^#^	1.6 (1.65)	3.27 (4.69)	2.52 (2.88)	5.56 (7.67)
Hospital mortality^*^	34,962 (3.1%)	80,301 (18.2%)	13,370 (10.2%)	17,610 (18.6%)
ICU mortality^*^	15,439 (1.4%)	59,911 (13.5%)	7,187 (5.5%)	11,170 (11.7%)

Variables marked with ^*^ are summarized using number (%); variables marked with ^#^ are summarized using median the interquartile range (IQR); all other variables are summarized using mean (sd).

Abbreviations: ICU, intensive care unit; LOS, Length of Stay.

The median percentage of patients ventilated was 33%. Broken down by ventilation type, ICUs had a median of 25% of stays invasively ventilated, and a median of 11% of stays non‐invasively ventilated. However, there was considerable variation around these numbers, with some ICUs ventilating or invasively ventilating a very large proportion of stays. Although, very high rates of use of non‐invasive ventilation alone were less common.

### 3.2 Model performance

The model's performance was evaluated based on predicting patients’ need for ventilation during their stay as well as their need for invasive or non‐invasive ventilation.

The overall model discrimination, evaluated by AUC, is shown in Figure 2. On the test data, the model provided AUC of 0.92, 0.94, and 0.83 for predicting any ventilation, invasive ventilation, and non‐invasive ventilation, respectively. Similarly, for the external test dataset, the model performance showed the AUC of 0.88, 0.90, and 0.82 for similar outcomes. The ROC curves for each predicted outcome on test data as well as external test data are also illustrated in Figure 3 and in the Appendix Figure 1, respectively.

### 3.3 Feature importance

To understand the information driving the model's predictions, we explored the most important features using SHAP values [14]. These yielded insights into the types of features contributing to model performance and the direction of association with the predicted outcomes.

Overall, patients with factors commonly generally associated with better health (such as younger age and lower BMI) were less likely to receive any ventilation during their stay (Figure 4a). High Glasgow Coma Scale (GCS) scores indicate lower severity of illness and were associated with lower probabilities of ventilation. Certain admission characteristics, such as the length of hospitalization prior to ICU admission; diagnoses of respiratory infection, heart failure, or coronary artery bypass graft (CABG); or admission from the operating room (OR) increased the likelihood the patient would be ventilated during their ICU stay. Conversely, admission from the emergency department (ED) was associated with a lower rate of ventilation. Higher respiratory rates and lower SaO2 values were associated with increased use of ventilation.

The features associated with the use of invasive ventilation were very similar to those associated with the use of ventilation in general (Figure 4b). High GCS scores and missing PF ratio measurements were associated with a decreased probability of receiving invasive ventilation, while an admission from the OR and an admission diagnosis of CABG were associated with an increased probability.

There were also commonalities in the features highly associated with the use of non‐invasive ventilation and those associated with a use of any ventilation, with three of the top five features in common (Figure 4c). Interestingly, GCS scores were not very predictive of the use of non‐invasive ventilation, while a missing PF ratio measurement was associated with a decreased probability of receiving non‐invasive ventilation. Admission diagnoses of respiratory infection, respiratory med, or CHF were highly predictive of the use of non‐invasive ventilation.

## 4 DISCUSSION

There have been several examples in the literature for predicting the need for ventilation. Shashikumar et al. [9] developed a neural network model to predict the need for mechanical ventilation. They used a dataset of nearly 3000 patients and achieved AUC of 0.89 on a validation dataset. Clark et al. [15] used logistic regression to study the need for prolonged mechanical ventilation for around 100 patients. APACHE III [16] provided a predictive model for the total duration of ventilation for patients at the onset of ventilation but without differentiating the ventilation type (invasive/non‐invasive). Roca et al. [8] provided a model to predict the probability of the use of nasal high‐flow therapy, a type of non‐invasive mechanical ventilation.

As compared to this previous work, the model presented in this study offers the advantages of being trained on a much larger, more comprehensive, and diverse dataset, providing high predictive accuracy with generalizability, and uniquely, providing predictions for the need for invasive and non‐invasive mechanical ventilation. In similar studies in predicting the use of any ventilation, our gradient boosting model outperformed similar works such as Shashikumar et al. (with **AUC = 0.92 **vs. AUC = 0.890), though validation is performed on their respective validation dataset.

We leveraged the eICU Research Institute dataset, comprising around 2.6 M patient stays across 350 hospitals in the United States between 2010 and 2019. Machine learning modelling on data from that many health systems and hospitals with potentially varying ventilation strategies can lead to improved model accuracy and generalizability.

The external test set comprised of all patient data from a single hospital not used during the model training allows robust assessment of the model as the distribution of patient data in this set does not necessarily follow the aggregate distribution of the training set that comprised patient stays from many hospitals. On this external test dataset, we achieved AUCs of 0.88, 0.90, and 0.82 for any ventilation, invasive ventilation, and non‐invasive ventilation, respectively. These values are indicative of model robustness, even for a new hospital that was not directly part of the training or test data distribution.

Used retrospectively, hospitals may use this model to benchmark their historical ventilation outcomes for their patient cohorts with other hospitals with similar mixes of patients. Investigating the important features using SHAP values revealed major contributors to ventilation outcomes. Features such as missing PF ratio measurements, high GCS scores, younger age, lower BMI, and lower PaCO2 are among indicators of a lower probability of ventilation need and possibly indicate that a patient in better health condition and is less likely to need mechanical ventilation. Interestingly, it was observed that having an admission source of OR increases the likelihood of invasive ventilation while conversely decreasing the likelihood of non‐invasive ventilation. Feature importance also revealed that older people are more likely to receive non‐invasive ventilation and less likely to receive invasive ventilation.

In terms of limitations, this work used data from 2010 to 2019, and thus the model does not include patients with COVID‐19, so it does not reflect ventilation practices for such patients. The study population are all within the United States and therefore might not generalize to other countries with different ventilation management strategies. The data comprised of nearly 50–50% female–male population ratio, however, may require further studies for racial, geographical, and wealth imbalances and other factors to further investigate biases in the model. With further work, the model can be optimized to be used prospectively as a decision support system, potentially providing decision support on patient ventilation strategies and improving resource management and patient care.

## 5 CONCLUSION

This work demonstrated a novel machine learning model for predicting patients’ need for mechanical ventilation as well as predicting the type of mechanical ventilation whether to be invasive or non‐invasive. The model was developed using a large, heterogeneous sample of hospitals with automated data collection of critically ill patients, with careful consideration of clinical workflow and documentation practices. Evaluation on model predictions on the patients’ ventilation status showed high accuracy when compared to actual ventilation status. To improve patient care quality, this model can be used retrospectively as a benchmarking tool for hospitals to compare their practices in ventilation strategies with the predictions made by our benchmark model.

## AUTHOR CONTRIBUTIONS

Mohsen Nabian and Emma Schwager led the analysis and wrote the first draft of this work. Ting Feng supported model creation. Xinggang Liu supported data creation and labelling. Louis Atallah, Omar Badawi, Pam Amelung, and Robin French performed clinical review, writing, and editing of the document.

## CONFLICT OF INTEREST STATEMENT

The authors declare no conflict of interest.

## FUNDING INFORMATION

This work was completely funded by Philips Healthcare.

## Supporting information

Supporting Information

## Data Availability

To construct the model detailed in the manuscript, we utilized a dataset possessed by Philips, encompassing approximately 4 million patients whose records span from 2010 to 2021. This dataset was sourced from clients of Tele‐ICU services based in the US, who, in a collaborative effort with Philips for research purposes, consented to provide their data. Due to the contractual obligations governing these associations, the comprehensive dataset cannot be publicly shared. Nevertheless, a subset comprising 200,000 patients has been disclosed as part of our partnership with MIT via PhysioNet. The dataset is accessible at: https://eicu‐crd.mit.edu/. Interested individuals can readily obtain this dataset and reproduce a significant portion of the outcomes detailed in this study.
