# Supplementary material for: Machine learning modelling for predicting the utilization of invasive and non‐invasive ventilation throughout the ICU duration
Source: Healthc Technol Lett. 2024 Feb 20;11(4):252–7. doi: 10.1049/htl2.12081 (PMC11294931; doi:10.1049/htl2.12081)
Supplement: Supplementary file 1 — Supporting Information [file HTL2-11-252-s001.docx]

# Supplement

**Appendix Table1: The physiological filtering ranges used.**Values within the normal range were retained. ‘(’ or ‘)’ indicates exclusion of the bound, while ‘[’ or ‘]’ indicates inclusion. For example, ‘(0, 1]’ indicates values > 0 and ≤ 1 were retained.

| **Measurement** | **Normal range** |  | **Measurement** | **Normal range** |
| --- | --- | --- | --- | --- |
| Total GCS score | [1, 15] |  | Blood white blood cell | [0, 500000] |
| Eye GCS score | [1, 4] |  | Blood sodium | (0, 250] |
| Verbal GCS score | [1, 5] |  | Blood potassium | (0, 100] |
| Motor GCS score | [1, 6] |  | Blood creatinine | [0, 50] |
| Unable or estimated GCS score | [1, 5] |  | Blood hemoglobin | (0, 100] |
| Systolic BP | [0, 300] |  | Blood albumin | (0, 100] |
| Diastolic BP | [0, 250] |  | Blood lactate | [0, 100] |
| Mean BP | [0, 270] |  | Arterial blood gas, pH | [6.5, 8] |
| Heart rate | [25, 250] |  | Arterial blood gas, PaCO2 | (0, 200] |
| Respiratory rate | [0, 100] |  | Arterial blood gas, PaO2 | (0, 650] |
| SaO2 | [50, 100] |  |  |  |

**Appendix Table2: A summary of the features used as inputs to the prediction model. (*) represents required features for the model.**

| **Feature** | **Summary measure** | **Categorized** |
| --- | --- | --- |
| Admission BMI (*) | NA | NA |
| Gender (1=Female)  (*) | NA | NA |
| Hours in hospital  prior to ICU admission (*) | NA | NA |
| meanBP  (*) | Mean, variance | No |
| systolicBP  (*) | Mean | No |
| diastolicBP (*) | Mean | No |
| Heart rate  (*) | Mean, variance | No |
| Respiration rate  (*) | Mean, variance | No |
| SaO2  (*) | Mean | No |
| Glucose (*) | Mean | No |
| WBC  (*) | Mean | No |
| Sodium  (*) | Mean | No |
| Creatinine  (*) | Mean | No |
| Hemoglobin  (*) | Mean | No |
| Admission source | NA | Observation, Acute Care or Floor, Floor, Unspecified, ED, Recovery Room, Other Hospital, ER, Other ICU, Direct Admit, Chest Pain Center, PACU, ICU, SDU, OR |
| Admission diagnosis | NA | Yes |
| GCS score | NA | Yes |
| pH | Mean | Yes |
| Lactate | Mean | Yes |
| Albumin | Mean | Yes |
| PaCO2 | Mean | Yes |
| PF ratio | Mean | Yes |


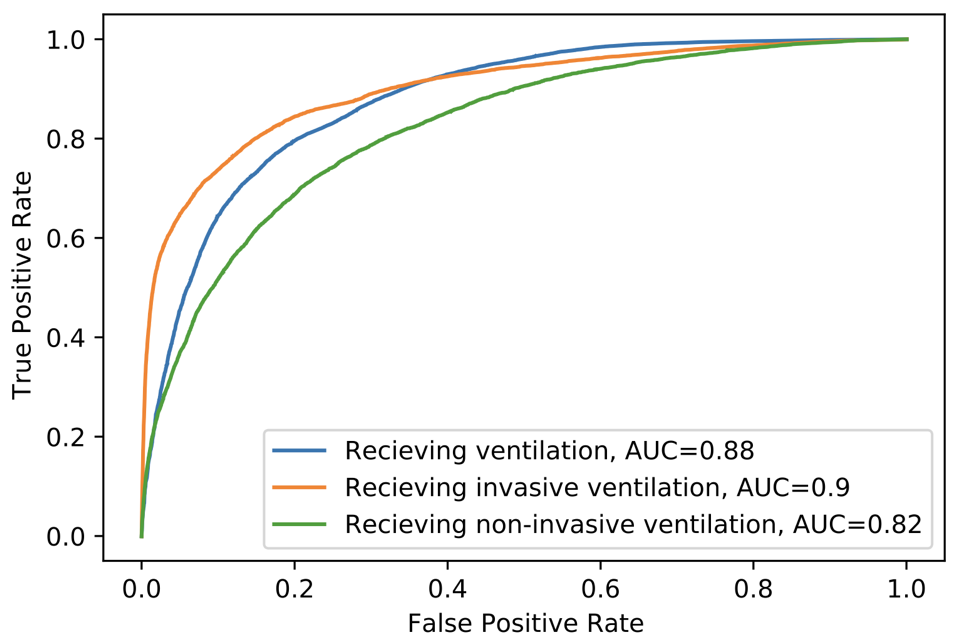


**Appendix Figure1: ROC Curve of External test data.** The model’s performance on external test data for identifying patients receiving any ventilation (blue), patients receiving any invasive ventilation (orange), or patients receiving any non-invasive ventilation (green).
